# Compound Dislocations1Read at the thirty-first annual meeting of the Medical Association of Central New York, at Auburn, October 18, 1898.

**Published:** 1899-01

**Authors:** Francis Eustace Fronczak

**Affiliations:** Buffalo, N. Y.


### COMPOUND DISLOCATIONS.[1]

By FRANCIS EUSTACE FRONCZAK, A. M., M. D., Buffalo, N. Y.

A DISLOCATION or luxation is a persistent, abnormal separation of the articular surfaces of adjoining bones. It differs from a sprain inasmuch as the latter is only a temporary and self-reduced luxation.

Dislocations may vary as to cause, in being traumatic, pathological or congenital, that is, they may either have been caused by external violence, whether it be direct or indirect, or by muscular action. Pathologically, a bone may be dislocated by a disease, as an abscess or a neophyte, destroying the ligaments and the joint tissue, and thus by a slow process and ultimately by muscular action, bringing about the displacement.

---

1. Read at the thirty-first annual meeting of the Medical Association of Central New York, at Auburn, October 18, 1898.

Finally, a congenital dislocation is one which is due to defective development of joint during intrauterine life, and hence, I would not call a congenital dislocation one, where the humerus or femur are pulled out of their sockets by the allwise midwife during delivery, as I have witnessed it already.

Dislocations are further subdivided as to their nature, as whether they are complete, partial or incomplete, generally called subluxations, complicated, *i. e.*, where there is a coëxistence of a fracture or other lesion besides the dislocation, a single and multiple, primary and secondary, recent and old or habitual, unilateral and bilateral, simple and compound dislocations. All these terms being self-explanatory, I shall not detain you in giving you their simple dictionary meanings.

I desire to speak in particular on compound as differentiated from simple dislocations. The latter is one where the articular surfaces do not communicate with the external air; the former is one where there is a communication of the cavity joint with outside air by means of a wound.

From statistics we find that dislocations in general are by far less in frequency than fractures; the proportion being, it is said, as one to ten. A compound dislocation, however, is still of greater rarity, it occurring only when great force is applied, that a bone is displaced from its normal relative position, and forced through the intervening tissue and the skin. Usually when so great a force is applied a fracture is the result and we do not have a compound dislocation, but a compound fracture, or a complicated luxation.

A compound dislocation may also be caused pathologically when, for instance, an abscess is permitted so to destroy the tissue surrounding articulating bones that one of them by slight force becomes disjointed. I have not seen or heard of a congenital compound dislocation, though I see no reason why even such an occurrence is not possible.

The symptoms of compound dislocation are usually the common symptoms of any dislocation. There is a deformity, that is, change in contour and attitude, which is always evident; there is pain, which may be very severe, especially if the nerve trunks are subjected to pressure, laceration, or are completely torn. The normal voluntary movement is very much restricted in certain directions. Another symptom is here which we do not find in other dislocations—namely, we see the damaged soft parts, a wound which may be either very small or quite extensive and sometimes the disjointed bone.

As far as diagnosis is concerned, it is not such an easy matter as is generally supposed. Much depends upon the symptoms and physical signs. In the first place, the rarity of the accident, in the second, the open wound and the probably exposed to view bone, are very misleading.

Well do I remember my first case of this kind. On seeing the protruding end of the styloid process of the radius, and especially the peculiar deformity, I could not help thinking of Colles's fracture. Such, however, was not the case. But I am anticipating.

The differential diagnosis between a compound fracture and a compound dislocation is chiefly determined by the absence of crepitus in the latter, though even this is not to be accepted as a dogma. Reëstablishment of a deformity after its correction, and the fact that dislocation once reduced generally keeps its place, are valuable diagnostic symptoms for differentiating purposes.

Prognosis, of course, is not as favorable in this kind of dislocation as it is in simple luxation. There is always some danger of suppuration and subsequent ankylosis, as we shall see later.

As to treatment we must necessarily adopt the ancient axiom of "Circumstances alter cases." Each such case must be a law unto itself. There may be a slight wound with hardly any possible infection; again, we may have to deal with an extensive injury of the soft parts and tissue surrounding the disarticulated bone and may fear suppuration. From my early university days, I always remember the maxim, "that the most important knowledge of surgery consists in the knowledge of attaining and maintaining absolute surgical cleanliness." The joint and wound is to be rendered as aseptic as possible, and then reduction accomplished. Sometimes a wound, no matter how slight, may prevent such rapid operative movements. It may even necessitate a larger opening, for the cavity joint may be contaminated and filled with particles of dirt and filth. This, of course, must be removed. We may even resort to excision of one of the bones in order to prevent ankylosis. Wound may or may not be closed at once, much depending on the circumstances. If the injury is extensive and was exposed to much contamination, a thoroughly good drainage and the packing of the cavity joint with iodoform gauze for a few days will be necessary. Of course, there is often a call for the performation of arthrotomy where suppuration follows compound dislocations, though, usually the open wound and the protruding bone are of sufficient pressure to bring about easy surgical attendance.

Passive motion is often resorted to, to prevent ankylosis, but it is said to do more harm than good; the stiffness that exists is only temporary, and aided by massage, hot and cold douches and the natural use of the limb, disappears gradually.

This, in short, covers my views on compound dislocations. In addition I desire to describe three of the several of this kind of accidents which I have seen during my practice:

CASE I.—Boy, F. P., age 16 years. Playing baseball; endeavored to steal a base by sliding. He threw his right hand forward, and as he fell the radius separated from the carpal bones; the wrist was turned backwards, the ligaments were torn and the styloid process of radius protruded through the tissue. This to all appearance looked like Colles's fracture. Examination with aid of skiograph, however, showed it to be a compound dislocation of the wrist. Reduction was accomplished, wound cleaned and closed, and the whole put in splints. Result was very satisfactory.

CASE II.—Boy, F. G., age 11 years. Was watering a horse. In some way or other the horse stepped on the boy's foot; result was that the first phalanx of the little toe became dislocated. I considered that as queer a *lusus naturæ* as ever occurred. The ligaments were simply torn, not a least bit of bone was broken or chipped and almost the entire bone was pushed through the skin and exposed to view. Reduction and aseptic dressing brought good recovery.

CASE III.—Man, T. T., age 37 years. Employed as a section hand on railroad. While carrying a rail, with several others, he fell on his left side, dislocating his left elbow, most probably having outstretched his left arm to save himself. The head of the radius and olecranon process of the ulna were pushed out through the tissues and the skin. The ligaments were torn and the bloodvessels ruptured and the man had almost bled to death when I saw him. Removal to hospital was refused, as also my request for another physician to aid me. The wound was cleaned, several vessels were ligatured, and after considerable labor the dislocation was reduced.

Provisions for drainage were made in the case and the wound dressed, the forearm being moved to right angle position. A few hours afterward I learned that a quack was called to review my work. All the bandages were removed and new maneuvers gone over. I heard of the case a short time ago, that the elbow was stiff and that he had been sick for months. No doubt, the amateur "bone setter" who was called after me, did not consider in the first place that cleanliness is of any value, and then, probably, the bones were dislocated again by the manipulation. At any rate suppuration must have followed with resulting ankylosis.

From this we see that where proper surgical methods are applied, we obtain satisfactory results. Failure to obtain these is usually due either to lack of knowledge of the true condition or to absence of proper care.

508 FILLMORE AVENUE.

